# Deep-learning methods for unveiling large-scale single-cell transcriptomes

**DOI:** 10.20892/j.issn.2095-3941.2023.0436

**Published:** 2024-02-05

**Authors:** Xilin Shen, Xiangchun Li

**Affiliations:** Tianjin Cancer Institute, Key Laboratory of Cancer Prevention and Therapy, Tianjin’s Clinical Research Center for Cancer, National Clinical Research Center for Cancer, Tianjin Medical University Cancer Institute & Hospital, Tianjin Medical University, Tianjin 300060, China

The rapidly evolving realm of single-cell transcriptomics offers vital new perspectives into the understanding of intra- and inter-cellular molecular dynamics governing development, physiology, and pathogenesis. Deep learning, a recent artificial intelligence advance with a promising application for big data, has demonstrated potential in the field of single-cell analysis^1^. Deep learning exhibits flexibility in extracting informative features from noisy, high-dimensional, single-cell RNA sequencing (scRNA-seq) data and enhances downstream analyses. We surveyed recent deep-learning methods that advance single-cell analysis and offer a glimpse into what the future holds.

## An overview of methods for analysing single-cell transcriptomes

The methods for single-cell analysis can be broadly categorized into statistical models and deep-learning methods.

Statistical modelling is a fundamental computational method in bioinformatics. Statistical modelling has been widely used in bulk tissue and single-cell transcriptome analysis. These methods include principal component analysis, canonical correlation analysis, and non-negative matrix factorization. Seurat^2^ utilized canonical correlation analysis to identify correlations among different datasets to construct mutual nearest neighbours for batch correction. MOFA+^3^ is built upon Bayesian group factor analysis to simultaneously capture variations across spatial and temporal covariates. In addition, MOFA+ handles shared and private sources of variations among different data modalities. scAI^4^ employs matrix factorization to integrate sparse single-cell expression and epigenetic profiles to aggregate sparse signals in similar cells, promoting consistent fusion with transcriptomic measurements.

In the realm of single-cell research, there is a paradigm shift from statistical models to deep-learning methods. Statistical models inherently face limitations when handling large-scale high-dimensional, non-linear, and complex structures in single-cell transcriptome data. Statistical models typically rely on prior assumptions and the modelling of linear relationships, which may manifest as an oversimplification when analysing single-cell transcriptome data. The complexity of single-cell transcriptome data involves non-linear relationships and a highly dimensional feature space, posing challenges for traditional statistical models in capturing the inherent complexity of the data. In addition, limited scalability poses challenges for statistical models when analysing the mounting volume of large-scale single-cell data over time^2^. Moreover, statistical models often require manual feature engineering. The characteristics of single cells can be influenced by multiple factors, which makes it difficult to comprehensively capture the characteristics through manually designed features. In this evolving field, a deep learning-based method offers researchers a powerful and flexible tool to better accommodate the diversity and dynamics of single-cell transcriptome data. The deep-learning method, through multi-layered non-linear transformations, adaptively learns features from the high-dimensional data without the need for predefined assumptions. The increased use of deep learning is aimed at surpassing the limitations of statistical models and comprehensively understanding and interpreting the intrinsic complexity of single-cell transcriptome data.

**Figure 1:**
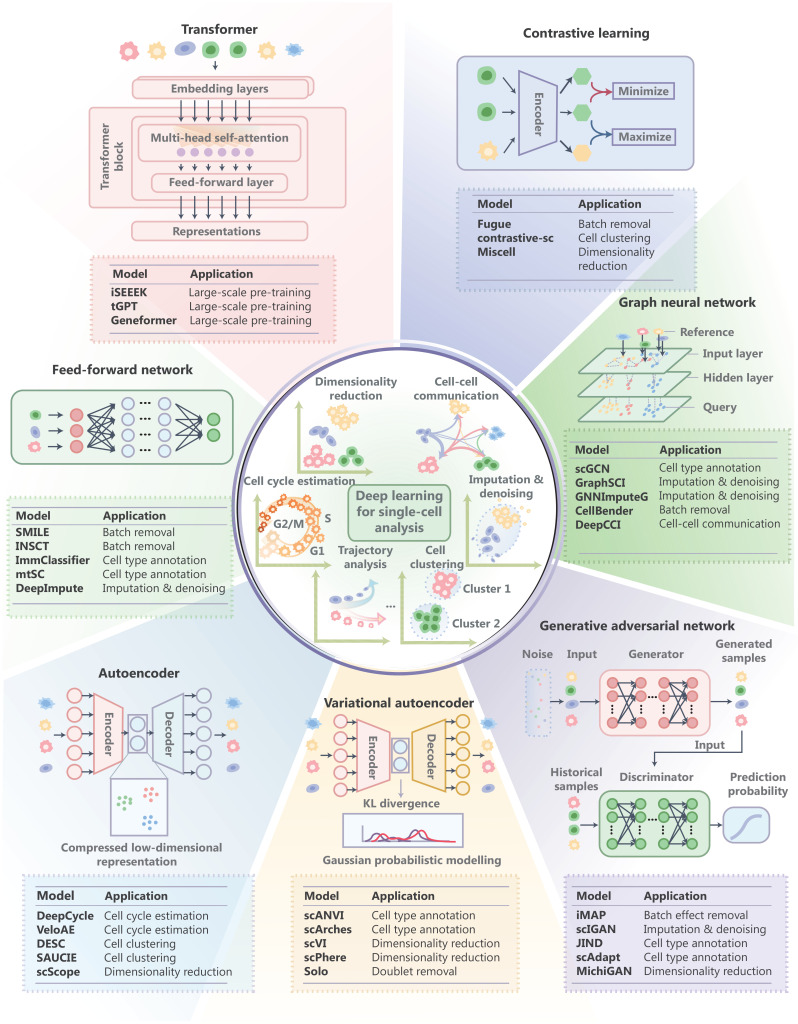
Summary of deep-learning model architectures and applications for single-cell analyses.

Deep learning, well known for remarkable advances in computer vision and natural language processing tasks, harnesses large-scale datasets to build models for downstream tasks. Deep learning has been successfully used to analyze single-cell data to improve our understanding of cellular processes^1^. **Figure 1** illustrates different types of fundamental deep-learning paradigms for single-cell transcriptome analysis.

Deep-learning methods can be categorized into unsupervised and supervised learning methods. In an unsupervised learning setting, the deep-learning model is trained on unlabelled data to uncover hidden patterns without explicit guidance. This paradigm is widely used for clustering cells, identifying cell subpopulations, and exploring the relationship between cell clusters and phenotypes^5,6^. Self-supervised learning represents a subtype of unsupervised learning, in which the model acquires knowledge by predicting specific elements of input data, frequently through the generation of pseudo-labels derived from the data^7^. Self-supervised learning is widely used in representation learning and dimensionality reduction^8^. The model is trained using labelled data in supervised learning. Supervised learning is used for tasks, such as cell type classification and denoising, in which labelled data are needed for training^9^. Semisupervised learning, as a subtype of supervised learning, can help bridge the gap in scenarios where labelled data are limited but abundant unlabelled data are available. Semisupervised learning is frequently used in cell type classification and gene-phenotype association^10^.

Deep-learning models commonly used in single-cell transcriptome deciphering include feed-forward networks (FFNs), autoencoders, variational autoencoders (VAEs), generative adversarial networks (GANs), graph neural networks (GNNs), and transformers^11–13^.

FFNs consist of multiple linear layers. FFNs are often used in supervised learning tasks. For example, DeepImpute^14^ utilizes a deep FFN to acquire insight into gene expression patterns and impute missing values in scRNA-seq data.

The deep autoencoder^11^ consists of an encoder and a decoder. The encoder is used to learn a compressed representation of the input. The decoder reconstructs the original input from this compressed representation. Applications of autoencoders extend across a broad spectrum of unsupervised learning tasks. For example, DESC applies an autoencoder to learn low-dimensional features of single-cell expression and performs clustering in the latent space to reduce the influence of batch effects iteratively^15^.

A VAE^12^ is a variant of an autoencoder. The advantage of VAEs over traditional autoencoders is the ability to generate data with uncertainty estimates. VAEs introduce Gaussian probabilistic modelling in the encoding process, allowing the model to capture the underlying probability distribution of the data. This leads to a more continuous and structured latent space, making it useful for tasks, such as data generation, interpolation, and denoising^5,16^. Single-cell variational inference (scVI)^5^ takes batch information into a VAE for batch correction. Single-cell annotation using variational inference (scANVI)^16^ is a semi-supervised adaptation of scVI. scANVI uses a mixture model to replace the Gaussian distribution for latent representations and overcomes the over-regularization issues when applied for cell state representation.

The GAN model^17^ consists of a generator and a discriminator that are trained in a competitive fashion. The generator learns to produce data that are indistinguishable from real data, while the discriminator learns to differentiate between real data and the fake data generated by the generator, leading to the generation of highly realistic synthetic data^17^. GANs are often used in generating realistic data, while autoencoders are often used in preserving feature representations in the latent space. Integration of multiple single-cell datasets by adversarial paired-style transfer networks integration (iMAP) combines the strengths of both autoencoders and GANs to achieve high-fidelity dimensionality reduction.

GNNs^13^ are designed to learn feature representations by propagating information through graph connections. GNNs excel at learning complicated associations by capturing dependencies between connected data points. Built upon GNNs, single-cell transcriptomics that uses a deep learning model with a weighted graph neural network (scDeepSort)^18^ constructs a cell-gene graph through pre-training on large-scale single-cell transcriptome data, leveraging the advantages of GNN to unveil complex cell relationships for downstream cell type annotation and elucidation of cellular interaction networks.

The transformer^19^ consists of self-attention mechanisms and feed-forward neural networks, enabling the transformer to learn contextual information of sequential data by capturing long-range dependencies across various positions. Transformer-based deep neural networks (DNNs) are continuing to revolutionize natural language understanding and computer vision.

Artificial intelligence is undergoing a paradigm shift with the rise in models trained on broad data that can be adapted to a wide range of downstream tasks. These models are referred to as foundation models. The transformer architecture underlies the success of foundation models due to high expressivity and scalability. Transformer-based DNNs are able to capture rich information from large-scale different types of datasets and generalize to new contexts. Because large-scale, single-cell data become increasingly accessible, transformer-based DNNs are being used for self-supervised pretraining with single-cell transcriptomes^20–22^. These pretrained models shed insight into gene–gene and gene-phenotype correlations. The pretrained models could serve as foundation models for subsequent fine-tuning in downstream tasks for cell-type annotation, gene-network analysis, and prediction of response for cancer patients who received immunotherapy treatment.

## Deep learning-based methods for large-scale single-cell transcriptomes

Advances in single-cell sequencing have led to the establishment of several public repositories housing large-scale, single-cell transcriptome data, such as the Human Cell Atlas (HCA), Single-Cell Expression Atlas, and Mouse Cell Atlas^23^. The HCA project^23^ is dedicated to curating trillions of single cells and constructing a comprehensive reference map of all human cells. Deep learning-based methods are well-suited for deciphering these exceptionally large-scale, single-cell transcriptome datasets.

Integration of large-scale, single-cell datasets is hampered by heterogeneity and sparsity of single-cell expression and batch effects^24^. INSCT^24^, a single-cell integration method designed for handling millions of cells, tackles batch effects by learning in a supervised manner using a triplet neural network to learn batch-aware cell representations by minimizing the distance between similar cells and maximizing the distance between dissimilar cells. Fugue^25^ is a self-supervised method capable of integrating super large-scale, single-cell transcriptomes from diverse sources by incorporating batch effects as a learnable parameter. Fugue can integrate all single-cell transcriptomes from HCA^25^, allowing for uncovering subtle variations across different biological states and tissue types. To address the inefficiency of t-distributed stochastic neighbor embedding (t-SNE) and uniform manifold approximation and projection (UMAP) in data visualization at the atlas-level scale, Cumulus^26^ provides a cloud-based solution for speeding up data visualization through training a deep feed-forward network. As described in **Table 1**, these methods come with specific aims to harness vast amounts of single-cell transcriptome data.

**Table 1:** Tools for analysing million-scale single-cell transcriptomes

Method		Learning strategy	Cell number	Task	Principle	Specificity	Ref.
**Statistic models**	**Scarf**	Graph-based t-stochastic neighbour embedding	4 million	Visualization	Graph-based neighbouring embedding and hierarchical clustering	Emphasizing rare cells and lineage trajectories	^27^
	**iNMF**	Online integrative non-negative matrix factorization	1.3 million	Data integration	Jointly decomposed inputs into shared and dataset-specific metagenes	Integrates datasets without needing the entire data during training	^28^
	**scMerge2**	Integrates single-cells in a hierarchical manner	11 million	Data integration	Hierarchical integration for local and global variations	Integrates incoming datasets without complete dataset availability during training	^29^
	**Seurat v5**	Dictionary learning	8.6 million	Data integration for multi-omic data	Decompose cells into multi-omics dictionary	Integrates data independent of single-cell omics measurements	^30^
**Deep-learning methods**	**Cumulus**	Supervised learning	1.3 million	Visualization	Learns project unseen cells with subsampling	Ensures a higher rate of sampling from rare cells	^26^
	**INSCT**	Semi-supervised learning	2.6 million	Data integration	Employs batch-aware triplet network to generate combined embedding space	Projects unseen single-cell data into pre-generated embeddings	^24^
	**Fugue**	Self-supervised learning	18 million	Data integration	Encoding batch information in unsupervised network	Maintains consistent memory usage across varying data magnitudes	^25^
	**SCALEX**	Unsupervised learning	4 million	Data integration	Applies VAE to project cells into a batch-invariant space	Incorporates incoming data without recalculating.	^31^
	**scPoli**	Semi-supervised learning	7.8 million	Data integration	Applying conditional VAE to regress batch effects	Explains sample and cell-level variations with sample embeddings	^32^
	**Concerto**	Self-supervised learning	10 million	Data integration for multi-omic data	Utilizes an asymmetric teacher-student architecture for cell pairing and batch separation	Pioneers multi-omics data integration	^33^
**Large-scale single-cell pre-training**	**iSEEEK**	Masked language modelling	11.9 million	Cell clustering, development trajectory, cell-cell communication	Leverages top 126 genes for each cell; predicts masked gene with bidirectional self-attention	Enables focused analysis and noise reduction in single-cell data; enhances contextual understanding	^20^
	**Geneformer**	Masked language modelling	29.9 million	Chromatin network and therapeutic targets inference	Leverages all genes within each cell; predicts masked genes using bidirectional self-attention	Fosters a comprehensive understanding of the cellular context; enhances contextual understanding	^22^
	**tGPT**	Auto-regressive modeling	22.3 million	Cell clustering, cell-phenotype, development trajectory, therapeutic targets inference.	Leverages top 64/126 genes for each cell; predicts the next gene based on previously generated genes	Enables focused analysis, noise reduction; suitable for single-cell data with temporal or positional order	^21^

The concept of large-scale, self-supervised learning has revolutionized natural language understanding and computer vision. Large-scale, self-supervised learning involves leveraging deep-learning models pretrained on large-scale general datasets and subsequent fine-tuning towards downstream tasks^22^. Representative pretraining models include masked and autoregressive language modelling. The advantage of these pretraining models lies in a capacity to absorb real-world insight from extensive unlabelled and high-dimensional data. Inspired by the success of large-scale pretraining in natural language understanding, transformer-based pretraining models have been developed for representing large-scale (10 million) single-cell transcriptomes, as exemplified by iSEEEK, Geneformer, and tGPT^20–22^. The input of these models is the sequence of gene symbols that are obtained by ranking the level of expression. iSEEEK^20^ and Geneformer^22^ use masked language modelling to learn cell representations. Specifically, 15% of genes were randomly masked during the training phase and the model was tasked to predict those masked genes by taking the unmasked genes in context, thereby enhancing the contextual understanding. The contextual relationships among genes learned by iSEEEK and Geneformer have proven to be useful in characterizing gene–gene and gene-phenotype associations. The difference between iSEEEK and Geneformer is the length of the input sequence. iSEEEK takes the top 128 expressed genes as input to avoid noise signals induced by low-expressing genes, while Geneformer uses the top 2048 expressed genes, which includes 93% of expressed genes, to avoid missing signals that are buried in low-expressing genes. The results showed that iSEEEK is robust with respect to the number of top-expressing genes^21^. tGPT^21^ was pretrained with autoregressive language modelling^34^. These rank-based methods are insensitive to batch effects, therefore providing more robust non-parametric feature representations for single-cell expression^21,22^. Single-cell transcriptomes are increasing exponentially. Therefore, the development of foundation models from these data has the potential to uncover biological principles governing cell development and transformation.

## Prerequisites and challenges of deep-learning methods

In the realm of analyzing large-scale, single-cell transcriptomic data, leveraging deep-learning methods hold promise, yet necessitates careful considerations and awareness of prerequisites. These considerations span multiple facets, influencing the efficacy and applicability of deep-learning models in deciphering the intricacies of single-cell biology. Rigorous data normalization is imperative to ensure that deep-learning models reliably extract meaningful information from raw data. Different deep-learning methods may require specific data normalization conditions. For example, the input expression vectors for iMAP are log-transformed transcripts per million (TPM)-like values^35^ and the tGPT model needs a count expression vector as the model input^21^. Moreover, researchers must grapple with strategies to apply suitable methods according to the data volume. For smaller datasets, leveraging techniques, such as transfer learning with the pre-trained model, are essential to mitigate the challenges posed by a limited sample size^21^. For large-scale datasets, advanced architectures, like contrastive learning^36^ and transformers^20^, may be more suitable for capturing complex patterns. Implementing appropriate strategies aligned with data volume ensures the robustness and generalization of the deep-learning models across different scales. Moreover, optimizing the model architectures and exploring lightweight alternatives become essential to strike a balance between computational efficiency and model performance. Techniques, such as model pruning and knowledge distillation, offer avenues to reduce model complexity without compromising predictive accuracy. Researchers must carefully assess the available computational infrastructure, considering factors, such as GPU availability and cloud computing resources, to determine the feasibility of implementing deep-learning approaches.

Single-cell data analysis often suffers from several challenges. The inherent noise and batch effects are the most significant challenges before data integrated analysis. Researchers should conduct rigorous batch correction to alleviate the impact of technical variations before applying deep learning methods. Addressing these challenges is crucial for enhancing the reliability and interpretability of the deep-learning models, particularly in the context of single-cell biology, in which data quality is paramount. In addition, single-cell transcriptomic data analysis faces inherent sparsity issues. The inherent sparsity arises from the nature of single-cell technologies, in which a substantial proportion of genes exhibit negligible levels of expression in individual cells, resulting in datasets dominated by zero values. This sparsity poses a hurdle for deep-learning models, making it arduous to discern meaningful patterns amid the noise, thereby impacting model performance and generalizability. Moreover, another notable challenge is the interpretability of models. Deep-learning models are often criticized for being “black boxes,” which makes it difficult to understand the biological rationale upon which the predictions are based. Balancing model complexity with interpretability becomes crucial, necessitating the incorporation of explanation methods. Researchers must explore techniques, such as attention mechanisms^19^, to unravel the features influencing model decisions. While attention mechanisms have not been extensively explored in single-cell analysis, the application in biology is gradually expanding. For instance, CLAM uses gate-based attention mechanisms to unearth context-independent morphologic pathology features in pathologic images^37^. WIT utilizes self-attention mechanisms to learn context-aware pathogenic features without any manual annotation^38^. In the realm of single-cell transcriptomics and deep learning integration, maintaining data quality and elucidating model outcomes remain pivotal. Only through a holistic consideration of noise, batch effects, sparsity, and explanations can the true potential of deep learning be realized in the context of single-cell biology.

In the dynamic landscape of single-cell analyzes, integrating multi-omics data, especially genomic and epigenomic data with single-cell transcriptomes, holds immense potential but is challenging. The primary hurdle lies in the inherent heterogeneity across data layers, demanding sophisticated computational methods to align disparate characteristics while preserving nuanced details. In the realm of multimodal integration, diverse omics modalities encounter distinctive challenges. For instance, the intrinsic sparsity in single-cell scRNA-seq data poses hurdles when integrating analysis. Single-cell assay for transposase-accessible chromatin using sequencing (scATAC-seq) exhibits sparsity and heterogeneity with noticeable diversity observed across different cell populations and gene loci. Similarly, single-cell methylation data are typically high-dimensional, involving millions of CpG sites per cell, rendering data processing and analysis more complex. In addition, the issue of imbalances between different modalities, in which some modalities may have fewer samples, is also a crucial consideration when integrating multimodal data, potentially influencing the integrated outcomes. In the realm of multi-omics data, challenges, such as batch effects between different omics layers, require specialized normalization techniques. Numerous alternative architectures have been suggested, each tailored to specific criteria, such as dropout and batch effect robustness, improved interpretability through disentanglement of latent factors and imputation of missing modalities through cross-modal translation^39^. Despite these challenges, multi-omics integration offers a comprehensive view of cellular processes, capturing the interplay between genetic variations, epigenetic modifications, and gene expression. For example, GLUE combines scRNA-seq and scATAC-seq for large-scale unpaired data analysis, unveiling expression quantitative trait loci and cis-regulatory interactions that single-omics approaches cannot explain^28^. Single-cell imputation protein embedding neural network (sciPENN) integrates cellular indexing of transcriptomes and epitopes by sequencing (CITE-seq) and scRNA-seq to reveal cellular heterogeneity in gene expression and functionality^40^. The holistic nature of multi-omics integration enhances biomarker discovery precision, identifying robust molecular signatures for accurate representation of cellular states. As technologies and methodologies advance, addressing these challenges will become increasingly feasible, opening new avenues for transformative insight into the complexities of single-cell biology^41^.

## Advancing single-cell biology research with deep learning in the future

Single-cell sequencing provides an unprecedented opportunity for the systematic investigation of cellular diversity and deciphering comprehensive delineation of the dynamics of single cells. The rapidly evolving field of deep learning has driven artificial intelligence (AI) research for biology, therefore addressing the biological challenges with AI.

Applications of AI for characterizing single-cell transcriptomes are still in the early stage. It is crucial to standardize the selection of deep-learning methods by taking into account statistical assumptions, the trade-off between scalability and accuracy, and the suitability of methods with analysis scenarios. Users need to choose the appropriate methods according to the characteristics of the data. Some deep-learning methods have specific statistical assumptions. For example, VAE requires an assumption of a Gaussian distribution, which does not hold for biased datasets with unbalanced cell types. In addition, different methods are suited to different tasks with different complexities. scVI performs better with large datasets and batch complexity. Seurat and DESC are recommended for batch correction of small datasets. Seurat and DESC will be valuable for a detailed evaluation of the relationship among different deep-learning methods and characteristics of single-cell data.

The growing volume of single-cell transcriptome data calls for the establishment of reference single-cell atlases across heterogeneous tissues from healthy individuals and patients with different diseases. Single-cell atlases include samples that span locations, laboratories, and conditions, leading to complex, nested batch effects in data. Joint analysis of atlas data requires reliable data integration. One of the main obstacles in atlas-level, single-cell integration is the detection of under- and over-correction. Under-correction can lead to incorrect biological interpretation because the observed differences between phenotypes can be due to batch effects. Over-correction can lead to the offset of true biological variation, especially when studying subtle shifts in the cellular state among different experimental conditions. It is imperative to establish a robust evaluation framework to measure batch effect correction and biological variation. This framework should encompass informative evaluation indices, as well as datasets with both quantifiable batch effects and biological variations in varying intensities.

AI research is undergoing a paradigm shift in computer vision and natural language processing. Pre-training methods that learn directly from raw text have revolutionized natural language processing. The emergence of GPT-4, boasting 1.8 trillion parameters and extensive training on 13 trillion tokens, expands the application of deep learning in various scenarios. With the amount of publicly available single-cell data continuing to expand, we will see a further paradigm shift in the integration of super large-scale transcriptomes into a fundamental model and transfer the knowledge into downstream tasks. Public databases host tens-to-hundreds of millions of single-cell transcriptomes, providing an ample resource to develop foundational models with increasing parameters for more comprehensive and effective interpretation of biological principles.

Additional data types derived from cells have accumulated in addition to transcriptomes. These data include genomic and epigenomic data generated by genome and epigenome sequencing. The integration of multi-omics data will open new avenues for a more comprehensive understanding of cellular dynamics across various hierarchies^28^. The establishment of large-scale pre-training frameworks for single-cell multi-omics analysis shows promise in revealing accurate regulatory relationships among different omics^41^. Large-scale pre-training frameworks for single-cell multi-omics is a rapidly improving content to explore deep representation learning by training multimodal deep neural networks. For example, contrastive language-image pre-training (CLIP)^42^ is pretrained on a substantial dataset of text and images, enabling CLIP to learn a shared understanding of language and vision for a variety of downstream tasks that link multi-omics. Leveraging the latest deep-learning multi-omics methods to learn shared feature representations of single-cell data holds great potential for a better understanding of genome-transcriptome interactions.

The development of single-cell analysis methods should not be the end goal but the application of single-cell analysis methods to enhance our current understanding of cell dynamics related to phenotypes. Current methods for associating cell types and sample conditions primarily focus on observing the proportions of different phenotypic samples within specific cell clusters. Exploring and characterizing subtle variations in phenotypes within identical cell types is imperative^43^. We believe there are still numerous uncharted paths and substantial opportunities to more deeply examine subcellular states and capture cell-to-cell variability of the homogenous cell populations.

Advances in deep-learning methods are poised to yield a deeper and better understanding of developmental processes, organismal functions, and disease development^44^. These methods will contribute to refining disease stratification, devising innovative therapeutic strategies, and advancing precision medicine.
